# Intelligent Dynamic-Enhanced Compensation for UAV Magnetic Interference

**DOI:** 10.3390/s25165059

**Published:** 2025-08-14

**Authors:** Zizhou Chen, Zhentao Yu, Cong Liu, Guozheng Wu, Jianwei Li, Dan Wang, Ye Wang, Yaxun Zhang

**Affiliations:** 1 Qingdao Innovation and Development Center, Harbin Engineering University, Qingdao 266500, China; czz@hrbeu.edu.cn (Z.C.); zhangyaxun@hrbeu.edu.cn (Y.Z.); 2 Navy Submarine Academy, Qingdao 266000, China; gaoxiaocong1121@163.com (C.L.); wgz980518@163.com (G.W.); lgm_jw@163.com (J.L.); wangdan-yn@sohu.com (D.W.); wangye2022@foxmail.com (Y.W.)

**Keywords:** magnetic interference compensation, Tolles-Lawson (T-L) model, dynamic-enhanced extended compensation model, GA-BP neural network, UAV aeromagnetic survey

## Abstract

Magnetic interference compensation is critical for enhancing the accuracy of unmanned aerial vehicle (UAV) magnetic anomaly detection. To address the constrained compensation performance of the conventional Tolles-Lawson (T-L) model, which stems from insufficient parametric dimensionality, this study proposes a dynamic-enhanced extended compensation model. The novelly introduced attitude angle and attitude angular rate-coupled features expand the parameter set from 18 to 34 terms, significantly enhancing the characterization of the magnetic field. To overcome the limitations of linear regression in modeling the nonlinear relationships inherent in small aeromagnetic datasets, we developed a genetic algorithm-optimized shallow backpropagation neural network (GA-BP). This network establishes high-precision correlations between the extended parameters and magnetic interference noise. Experimental results demonstrated that the proposed model effectively captured the coupling characteristics between dynamic flight attitudes and the interference field, leading to significant gains in key performance metrics. This approach provides novel optimization pathways for anti-interference capabilities in airborne detection systems, offering substantial practical value for enhancing UAV aeromagnetic surveys.

## 1. Introduction

Unmanned aerial vehicle (UAV) platform, also known as unmanned aircraft, is an aircraft that accomplishes flight missions via autonomous flight control technologies. It is capable of autonomous flight, mission execution, and self-protection. Equipped with diverse magnetic sensors and devices based on the requirements, UAVs are extensively used in military reconnaissance [1], environmental monitoring [2], geological exploration [3], aerial photography [4], and other magnetic-related fields. However, the ferromagnetic materials of UAVs can generate interfering magnetic fields that affect sensor measurements during flight due to environmental factors. Thus, compensating for these magnetic interferences to enhance the accuracy of sensor measurements is crucial in UAV-based magnetic surveys.

In 1960, Tolles and Lawson first established the T-L magnetic interference model [5], classifying magnetic interference into constant, induced, and eddy-current types based on its nature and causes. Based on this model, Leliak [6] developed a set of FOM flight rules. UAVs perform specific maneuvers to generate magnetic interference fields, which are then used to solve for magnetic interference compensation coefficients. However, the parameters in the model are interdependent, leading to multicollinearity and instability in the compensation coefficients [7]. In 1980, Leach reformulated the T-L model solution as a linear regression problem, proposing ridge regression for coefficient estimation [8]. Bicke applied the small signal method to achieve more stable solutions with reduced multicollinearity [9]. Wu et al. used principal component analysis (PCA) to mitigate multicollinearity in the T-L model [10].

The linear-regression-based aeromagnetic compensation method has insufficient fitting function ability. In contrast, neural networks possess stronger fitting capabilities [11,12]. In 1993, Williams [13] first integrated neural networks with aeromagnetic compensation, establishing the first intelligent aeromagnetic compensation model. However, due to overfitting, this model was not widely applied, yet it offered new research ideas. In 2020, Yu [14] utilized an autoencoder network for aeromagnetic compensation. Although utilizing the T-L model parameters as network inputs enhanced compensation accuracy to some extent, limitations persisted due to the inadequate incorporation of interference sources within the input parameters. In 2021, Emery [15] developed a magnetic interference compensation model based on neural networks for the F-16. The model incorporated compensation parameters from the T-L model as input features and introduced voltage and ampere sensor data as additional inputs. In 2022, Yu et al. [16] used the 18 coefficients of the T-L model as inputs for a Res-BP residual neural network, effectively alleviating the gradient vanishing problem in traditional neural networks and improving compensation accuracy. However, Res-BP networks were primarily designed to mitigate gradient vanishing in deep architectures with large-sample datasets to maintain network stability, rendering this structure suboptimal for small-sample scenarios due to inherent limitations. Also in 2022, Jiao et al. [17] employed a compressed and accelerated neural network, using simplified T-L model coefficients as inputs and pre-training weights with transfer learning to reduce model convergence time and enhance compensation effects. Additionally, in 2022, Zhou [18] conducted numerical simulation experiments on aeromagnetic data magnetic interference using drones and proposed a radial basis function (RBF) artificial neural network (ANN) algorithm for aeromagnetic data compensation. In 2024, Ma et al. [19] combined attention mechanisms with the T-L model using deep learning, achieving better compensation accuracy and performance than classical linear regression and data-driven methods. Meanwhile, Favour [20] adopted a physics-informed method, using T-L model coefficients for compensation and liquid time-constant networks (LTCs) to remove complex noise signals from aircraft magnetic sources, significantly improving compensation accuracy. All these methods use the coefficients of the basic T-L model as neural network inputs. While they have improved compensation accuracy, the limited number of coefficients restricts the inclusion of interference factors. Furthermore, for aeromagnetic data with a small amount of data, increased network complexity heightens overfitting risks. For limited aeromagnetic datasets, heightened network complexity risks overfitting. Augmenting input parameters while employing shallow architectures effectively enhances compensation performance by expanding interference representation and simplifying structure.

Starting from the generation principle of the UAV interference sources, eddy-current interference items have more influencing factors compared to fixed and induced magnetic fields. Based on the improved compensation flight circle data from reference [21], we dynamically extended the eddy-current interference items in the conventional T-L model using enhancement functions. The derived coefficients were used as inputs for a shallow GA-BP neural network to train the model for magnetic interference compensation. The results showed that this method improves compensation accuracy.

## 2. Principle

### 2.1. T-L Model

**Figure 1:**
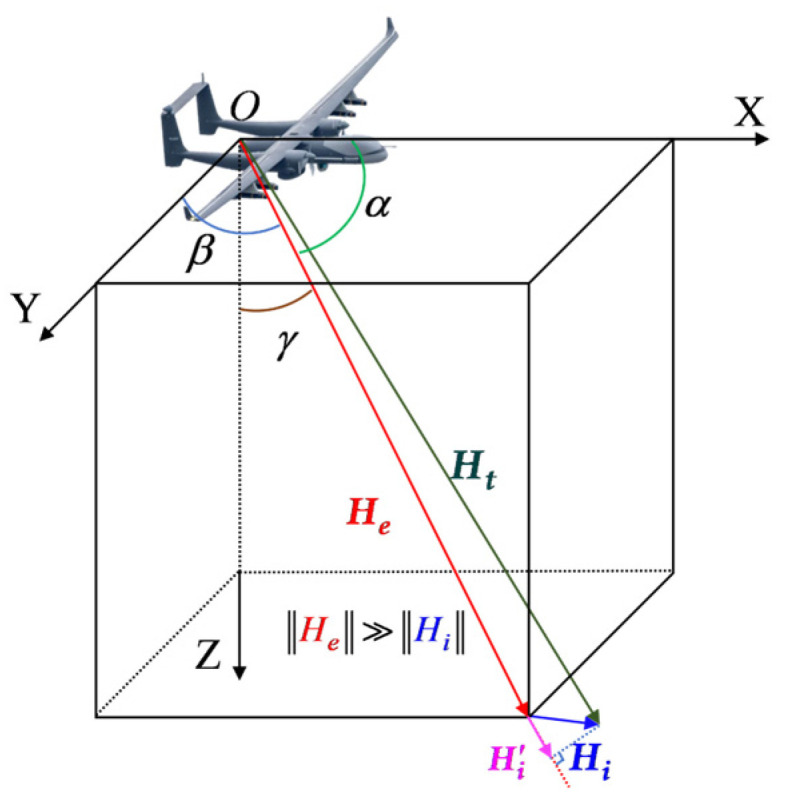
Coordinate system of the aeromagnetic compensation interference model, with disturbance field $H_{i}$ projected onto the geomagnetic field direction $H_{i}^{\prime }$.

As shown in Figure 1, prior to aeromagnetic compensation, a right-handed orthogonal coordinate system was established according to the T-L model convention [5], with the UAV center of mass as the origin (O). The X-axis is aligned with the fuselage longitudinal axis (positive forward in the flight direction), the Y-axis extends perpendicularly to the fuselage symmetry plane (positive toward the starboard wing), and the Z-axis completes the right-hand rule (positive downward normal to the Earth’s surface). The total magnetic field $H_{t}$ measured by the optically pumped magnetometer (OPM) comprises the geomagnetic field $H_{e}$ and the aircraft-induced interference $H_{i}$, expressed as

(1) $$H_{t}=H_{e}+H_{i},$$

Given the significant disparity in field magnitudes (‖$H_{e}$‖ ≫ ‖$H_{i}$‖), the OPM output effectively represents the projection of ${H_{i}}^{\prime }$ onto the direction of $H_{e}$, characterized by direction cosines cos α, cos β, and cos γ. α, β, and γ denote the angles between $H_{e}$ and the X-, Y-, and Z-axes, respectively.

The T-L model classifies aircraft interference into the following three components: permanent magnetization originating from ferromagnetic materials, induced magnetization proportional to the geomagnetic field intensity, and eddy-current effects dependent on the time derivatives of $H_{e}$. For typical UAV ferromagnetic components (e.g., motors and actuators), the relative permeability ranges between 200–500. Given the Earth’s magnetic field strength, this ensures the linear approximation (μ_r_ ≈ constant) holds. Consequently, the interference field is formulated as the superposition of these components:

(2) $$\begin{array}{l} {H_{i}}^{\prime }=H_{per}+H_{edd}+H_{inc}\\ \;=\sum_{i=1}^{3}p_{i}u_{i}+H_{e}\sum_{i=1}^{3}\sum_{j=1}^{3}b_{ij}u_{i}u_{j}^{\prime }+H_{e}\sum_{i=1}^{3}\sum_{j=1}^{3}a_{ij}u_{i}u_{j} \end{array},$$

Here, ${H_{i}}^{\prime }$ represents the projection value of the disturbance field onto the geomagnetic field direction, $H_{per}$ represents the projection value of the constant field onto the geomagnetic field direction, $H_{edd}$ represents the projection value of the eddy-current field onto the geomagnetic field direction, and $H_{inc}$ represents the projection value of the induced field onto the geomagnetic field direction. $p_{i}$ (i = 1, 2, 3) are the three-component values of the projection of the permanent magnetic field in the body coordinate system. $a_{ij}$ (i, j = 1, 2, 3) are the induction coefficients of the induced magnetic field. $b_{ij}$ (i, j = 1, 2, 3) are the eddy-current interference coefficients. $u_{i}$ and $u_{j}$ (i, j = 1, 2, 3) are the direction cosines calculated from the magnetic data obtained via a three-axis fluxgate $H_{i}$ (i = 1, 2, 3). $u_{j}^{\prime }=du_{j}/dt$ (i, j = 1, 2, 3) are time derivatives of the direction cosines. They reflect the rate of change in the geomagnetic field’s direction cosines due to aircraft attitude variations and serve as key parameters in eddy-current magnetic field modeling. The calculation formulas are as follows:

(3) $$u_{i}=\cos\alpha_{i}=H_{i}/\sqrt{\sum_{i=1}^{3}{H_{i}}^{2}},$$

Here, $\alpha_{i}$ (i = 1, 2, 3) respectively represents the angles α, β, and γ between the $H_{e}$ and the three axes of the body coordinate system.

For the same aircraft, the permanent magnetic field is constant. The physical mechanism of the induced magnetic field is $\left|B\right|=\mu_{\mathrm{rel}}(H)\mu_{0}\left|H\right|$ that the initial induced magnetic field is the external magnetic field multiplied by a magnetic induction coefficient. $\mu_{\mathrm{rel}}(H)$ represents relative permeability as a function of field strength H, accounting for nonlinear material properties. After a series of inductions, the magnetic induction process reaches equilibrium quickly. The eddy-current magnetic field is generated by the metal components of the aircraft cutting the magnetic field lines during flight. For ferromagnetic UAV components, $\mu_{\mathrm{rel}}(H)$ exhibits nonlinear behavior with H, necessitating field-dependent modeling in eddy-current calculations. According to Faraday’s law of electromagnetic induction and the Biot–Savart law, the basic principle of the eddy-current magnetic field is as follows:

The induced electromotive force (EMF) generated when a circular conductor on the UAV (with a circumference of L=2πr) cuts through the Earth’s magnetic field $H_{0}$ at a velocity of $\lambda_{m}\omega \sin\lambda \cos\omega t$ is

(4) $$\varepsilon =-\frac{d\Phi }{dt}=-\mu_{0}H_{0}Lv\sin\lambda =-\mu_{0}H_{0}L\lambda_{m}\omega \sin\lambda \cos\omega t$$

Here, the angle λ between the conductor’s motion direction and the Earth’s magnetic field represents the drone’s attitude angle, where $\lambda_{m}$ is the maximum angular displacement, ω is the angular velocity, and $\mu_{0}H_{0}$ is the magnetic flux density of the Earth’s field $B_{0}$.

The magnitude of the eddy current is limited by the conductor’s resistance R:

(5) $$I=\frac{\left|\varepsilon \right|}{R}=\frac{\mu_{0}H_{0}L\lambda_{m}\omega \sin\lambda \cos\omega t}{R}$$

The magnetic field generated by the eddy current I at point M on the axis (located at a distance x from the center of the circle) is given by

(6) $$dB=\frac{\mu_{rel}(H)\mu_{0}\cdot \mu_{0}I}{4\pi }\cdot \frac{r^{2}dl}{{\left[r^{2}+{\left(x+l\right)}^{2}\right]}^{3/2}}$$

Here, dl denotes the infinitesimal length element of the circular conductor. The integral over the circumference of the ring (with total length L=2πr) yields

(7) $$B=\frac{\mu_{rel}(H)\mu_{0}\cdot \mu_{0}Ir^{2}}{4\pi }\int_{0}^{L}\frac{dl}{{\left[r^{2}+{\left(x+l\right)}^{2}\right]}^{3/2}}$$

Substituting the expression for the eddy current I into Equation (1) yields

(8) $$\begin{array}{ll} B & =\frac{\mu_{rel}(H)\mu_{0}\cdot \mu_{0}r^{2}}{4\pi }\cdot \frac{\mu_{0}H_{0}L\lambda_{m}\omega \sin\lambda \cos\omega t}{R}\int_{0}^{L}\frac{dl}{{\left[r^{2}+{\left(x+l\right)}^{2}\right]}^{3/2}}\\ & =\lambda_{m}\omega \sin\lambda \cos\omega t\cdot \frac{r^{3}\mu_{rel}(H)\mu_{0}\cdot \mu_{0}^{2}H_{0}}{2R}\int_{0}^{L}\frac{dl}{{\left[r^{2}+{\left(x+l\right)}^{2}\right]}^{3/2}} \end{array}$$

**Figure 2:**
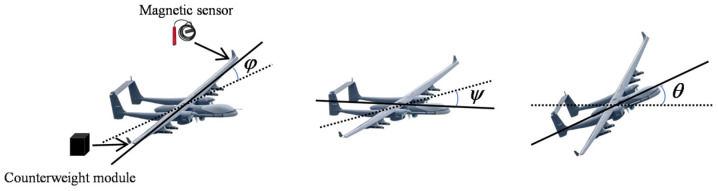
Attitude angles of UAV under inertial navigation system.

For aircraft-mounted fixed-position coils, $\frac{r^{3}\mu_{rel}(H)\mu_{0}^{3}H_{0}}{2R}\int_{0}^{L}\frac{dl}{{\left[r^{2}+{\left(x+l\right)}^{2}\right]}^{3/2}}$ is constant. The eddy-current-generated magnetic interference thus depends on the amplitude $\lambda_{m}$, the UAV’s attitude angles (φ, ψ, θ), and its angular velocities ($\varphi^{\prime }$, $\psi^{\prime }$, $\theta^{\prime }$). These factors are incorporated into the T-L model for eddy-current magnetic field enhancement. As shown in Figure 2, the UAV’s attitude, including φ, ψ and θ is obtainable via the inertial navigation system.

As the magnetic data used were obtained from the improved flight coils in reference [21], there were no ψ data. Therefore, only the φ, θ, and their related terms were used as additional data. Consequently, the enhanced T-L model becomes

(9) $$\begin{array}{l} {H_{i}}^{\prime }=H_{per}+H_{edd}+H_{inc}\\ \;=\sum_{i=1}^{3}p_{i}u_{i}+H_{e}\sum_{i=1}^{3}\sum_{j=1}^{3}a_{ij}u_{i}u_{j}+\left(f\left(\varphi ,\theta ,\varphi^{\prime },\theta^{\prime }\right)+H_{e}\sum_{i=1}^{3}\sum_{j=1}^{3}b_{ij}u_{i}u_{j}^{\prime }\right) \end{array}$$

Here, $f\left(\varphi ,\theta ,\varphi^{\prime },\theta^{\prime }\right)$ is defined as the dynamic enhancement function, and is expressed as follows:

(10) $$\begin{array}{ll} f\left(\varphi ,\theta ,\varphi^{\prime },\theta^{\prime }\right)= & k_{1}\varphi +k_{2}\theta +k_{3}\theta \varphi +k_{4}\varphi^{2}+k_{5}\theta^{2}+k_{6}\varphi^{2}\theta +k_{7}\theta^{2}\varphi +k_{8}\varphi^{2}\theta^{2}\\ & +k_{9}\varphi^{\prime }+k_{10}\theta^{\prime }+k_{11}{\varphi^{\prime }}^{2}+k_{12}{\theta^{\prime }}^{2}\\ & +k_{13}\cos\varphi +k_{14}\cos\theta +k_{15}\sin\varphi +k_{16}\sin\theta \end{array}$$

When the UAV performs a single maneuver, minor involuntary movements in other degrees of freedom are inevitable. The coupling of the angle powers reflects the impact of multi-DOF (degrees of freedom) coupling on the eddy-current magnetic field. The higher-order terms for the angles and angular velocities capture nonlinear effects. The trigonometric terms (sin θ, cos φ) accurately reflect how conductor orientation affects the direction of induced electromotive force, in line with Faraday’s law. Based on this, the expanded T-L model can be simplified as:

(11) $$H_{i}=MC$$

Here, **M** is a matrix composed of direction cosines, combinations of direction cosines with geomagnetic field values, combinations of direction cosines and their time derivatives with the geomagnetic field, Euler angle-related terms, and angular velocity-related terms. **C** is the matrix of coefficients to be determined, with 34 coefficients to solve for after simplification.

The equation is solved using the least squares method, with the solution formulated as $C={\left(M^{T}M\right)}^{-1}M^{T}H_{i}$. Substituting the solution back into the equation yields the compensated interference field.

In addition, after obtaining the compensated interference field, evaluation metrics are needed to assess the compensation effectiveness. Hardwick introduced the standard deviation (STD) and improvement ratio (IR) for this purpose in 1984 [22]. The STD is the root mean square of the compensated results after bandpass filtering. The IR is the ratio of the STD of the pre-compensation signal to that of the post-compensation results after bandpass filtering. A higher IR indicates better compensation. The formulas are as follows:

(12) $$STD\left(H\right)=\sqrt{\frac{1}{n}\sum_{i=1}^{n}{\left(H_{i}-\bar{H}\right)}^{2}}$$

(13) $$IR=\frac{STD\left(H_{u}\right)}{STD\left(H_{c}\right)}$$

Here, $\bar{H}$ represents the mean value of n magnetic field scatter data, $H_{u}$ represents the standard deviation of the magnetic field before compensation, and $H_{c}$ represents the standard deviation of the magnetic field after compensation.

### 2.2. Genetic Algorithm-Optimized BP Neural Network Model

**Figure 3:**
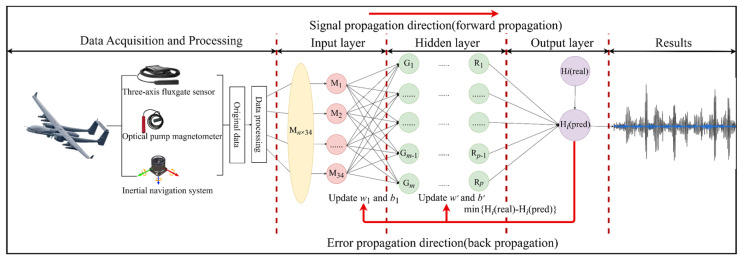
Structural diagram of aeromagnetic compensation based on the BP neural network.

The BP neural network, based on error backpropagation, establishes input–output nonlinear mappings through a layered structure of fully connected input, hidden, and output layers. The structure of the BP neural network for aeromagnetic compensation is shown in Figure 3.

The BP neural network executes forward propagation from input through hidden to output layers and backward error propagation, where the iterative gradient descent updates of inter-layer weights and biases minimize output error. The specific principles are as follows:

(1) Data Preparation and Parameter Initialization

Data preparation required feature extraction and [0, 1] normalization to accelerate convergence. Data were split into training (60–80%), validation (10–20%), and test sets (10–20%) for parameter updates, hyperparameter tuning, and evaluation. During parameter initialization, we initialized the weight matrices, bias terms, and learning rate, and confirmed the number of nodes in the input, hidden, and output layers. The relationships among these parameters are as follows:

(14) $$p=\sqrt{m+n}+k$$

Here, m is the number of input layer nodes, p is the number of hidden layer nodes, and n is the number of output layer nodes. k is an adjustment constant, typically an integer between 1 and 10.

(2) Data Feedforward Process

After data preprocessing and initialization, samples were propagated through the network via weighted connections between layers, computed as

(15) $$Y_{j}=\sum_{i=0}^{m-1}\left[w_{ij}f\left(Y_{j-1}\right)+b_{j}\right]$$

Here, $w_{ij}$ represents the weight between node i and node j, $b_{j}$ represents the bias term of node j, $Y_{j}$ represents the function value of output node j, $f\left(x\right)$ represents the activation function, and $f\left(Y_{j-1}\right)$ represents the function value of input node j after being processed by the activation function. In this paper, tanh is selected as the activation function, and its expression is as follows:

(16) $$\tanh\left(x\right)=\frac{e^{x}-e^{-x}}{e^{x}+e^{-x}}$$

(3) Error Backpropagation Process

The backpropagation process starts from the output layer and calculates the error. Gradient descent is employed to update the weights. The error function is as follows:

(17) $$error\left(w,b\right)=\frac{1}{2}\sum_{j=0}^{j-1}\left(y_{j}-n_{j}\right)$$

Here, $y_{j}$ is the output value of the neural network, and $n_{j}$ is the actual value.

Gradient descent expands the error function using a Taylor series, discards high-order terms, and retains first-order terms and constant terms. The functional expression is as follows:

(18) $$\delta_{n+1}=\delta_{n}-\eta \frac{\partial error\left(w,b\right)}{\partial \delta_{n}}$$

Here, δ represents the parameter values (including w and b) after n updates, η represents the learning rate, and the update iteration process for the parameters is as follows:

(19) $${\left(\omega_{ij}\right)}_{n+1}={\left(\omega_{ij}\right)}_{n}-\eta \frac{\partial error(w,b)}{\partial \omega_{ij}}$$

(20) $${\left(b_{j}\right)}_{n+1}={\left(b_{j}\right)}_{s}-\eta \frac{\partial error(w,b)}{\partial b_{j}}$$

(4) Iterative Update Process

**Figure 4:**
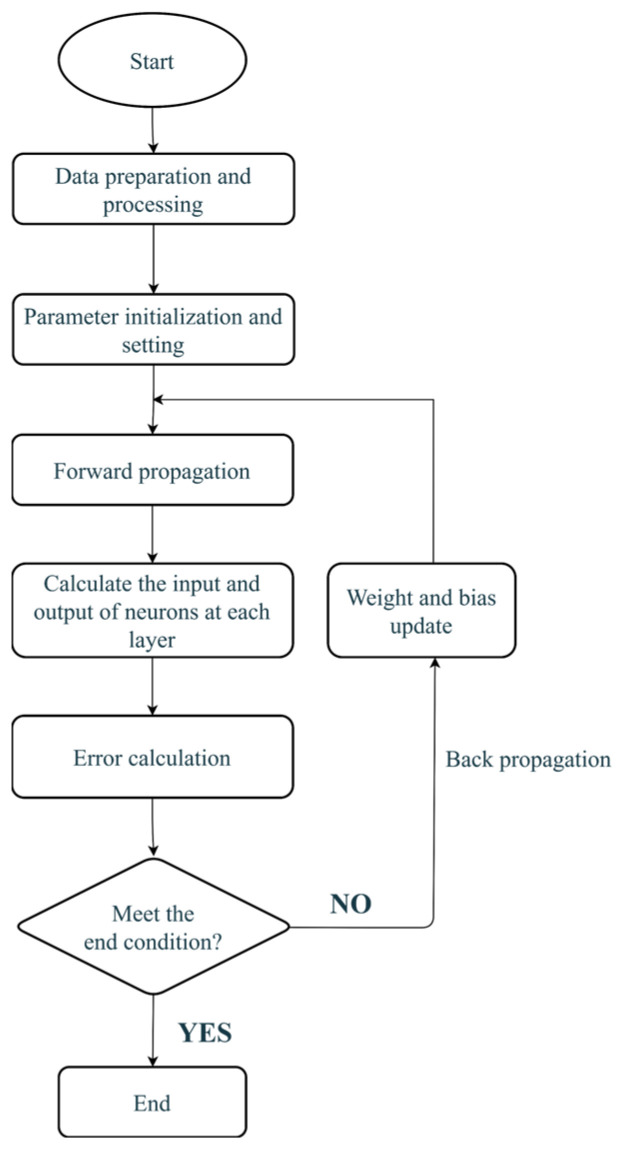
BP neural network flowchart.

The network iteratively updates inter-layer weights and biases per parameter update cycle until the prediction error falls below the present threshold. The flowchart of the BP neural network is shown in Figure 4.

Genetic algorithms (GA) optimize BP neural networks by evolving weight and bias parameters through selection, crossover, and mutation. The process begins by defining the fitness function minimizing the mean squared error and initializing a population encoded with network parameters. Selection favors high-fitness individuals, while crossover and mutation generate diverse offspring by exchanging or perturbing parameters. The population iteratively updates until meeting termination criteria, including max iterations or fitness convergence. This approach can efficiently explore the parameter space, enhancing BP network performance.

**Figure 5:**
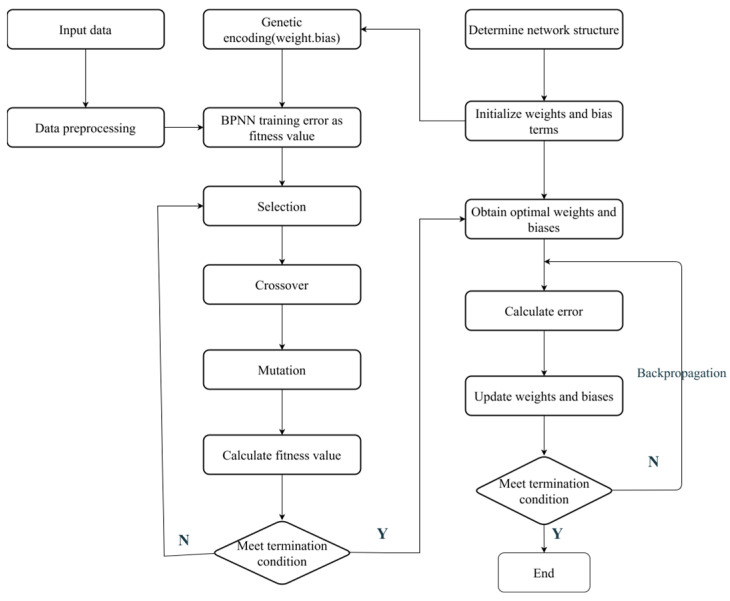
GA-BP algorithm flowchart.

Based on the above analysis, the flowchart of BP neural networks using genetic algorithms is shown in Figure 5.

## 3. Results

### 3.1. Experimental Data Acquisition and Preprocessing

To compensate for UAV’s magnetic interference, it was necessary to obtain magnetic interference data of the UAV through compensation flights. In traditional compensation flights, the UAV flies in the order of north, east, south, and west. In each direction, three sets of maneuvering actions (pitch, roll, yaw) are performed respectively. Each set of maneuvers lasts approximately 5–8 s, with 5 s of level flight interspersed between each set, ultimately forming a square flight framework. In this paper, however, the improved compensation flight loop flight rules proposed in [21] were used to acquire compensation data. The modified scheme in the literature eliminated yaw maneuvers and optimized the flight path, resulting in a flight trajectory composed of two quadrilaterals, and verified the feasibility and reliability of the modification.

**Figure 6:**
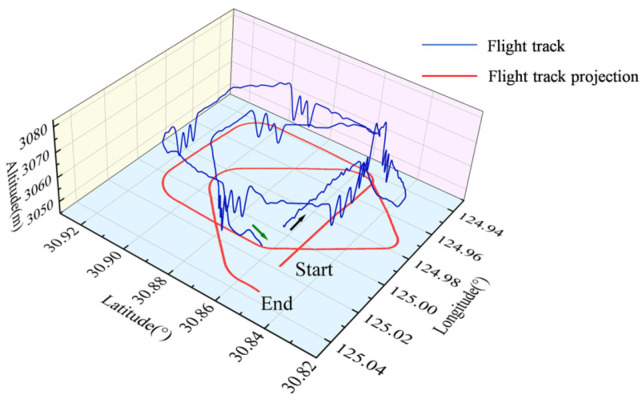
Flight trajectory diagram of the UAV with compensation. The red curve represents flight track projection.

The compensation flight trajectory diagram of this experiment is shown in Figure 6. The black arrow represents the starting direction of the compensation flight, and the green arrow represents the ending direction of the compensation flight. The red curve represents the projection of the UAV’s 3D track map in the horizontal direction. Two sets of compensation flight data, comprising feasibility validation data and actual test data, were acquired through compensation flight tests.

**Figure 7:**
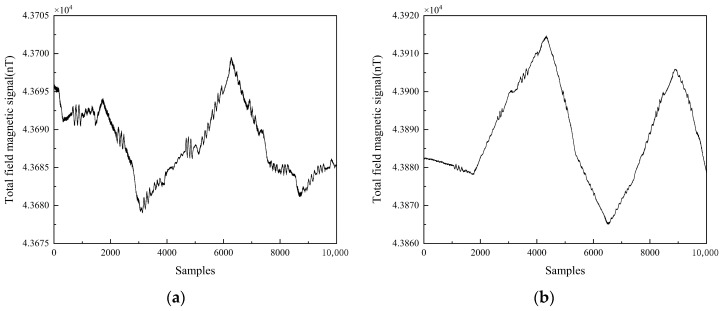
Total field magnetic signal: (**a**) Feasibility validation plot; (**b**) Formal test plot.

**Figure 8:**
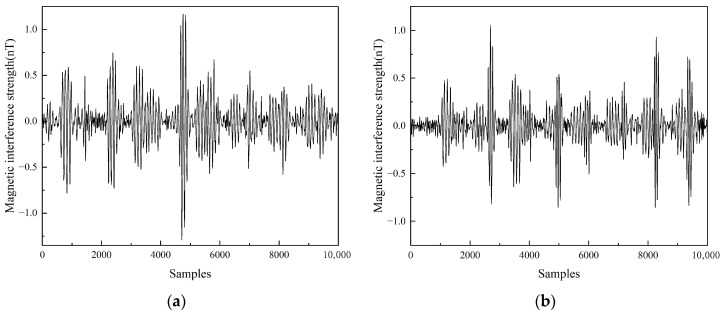
Magnetic interference in the compensation flight: (**a**) Feasibility validation plot; (**b**) Formal test plot.

The quality of data preprocessing significantly influenced the effectiveness of the magnetic interference compensation algorithms. The total magnetic field signal is shown in Figure 7, which represents the superposition of the geomagnetic field and magnetic interference signals. To isolate pure interference, signal essential for compensation calculations, this study implemented a Butterworth bandpass filter characterized by its flat passband response. This filter design effectively attenuated the low-frequency geomagnetic components that exhibit substantial amplitude yet gradual variations, while simultaneously maintaining the integrity of high-frequency interference elements. The resulting UAV magnetic interference signal presented in Figure 8 conclusively verified the method’s capability to distinguish and extract target signals from overwhelming geomagnetic noise.

### 3.2. Magnetic Interference Compensation Experiment

**Figure 9:**
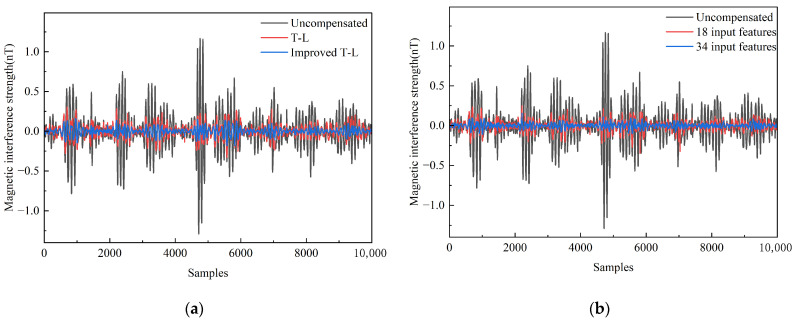
Feasibility validation results. (**a**) Result of feasibility verification for linear regression method; (**b**) Result of feasibility verification for neural network method (BP neural network).

To verify the feasibility of the dynamic-enhanced model incorporating expanded eddy-current terms, validation was performed using both conventional linear regression and neural network approaches. Firstly, magnetic interference compensation was implemented using the traditional least squares method for coefficient solving. Figure 9a compares the compensation performance between the 18-term and 34-term coefficient sets. Subsequent neural network verification was performed by contrasting two input feature configurations: traditional T-L 18-term magnetic interference coefficients versus dynamics-expanded 34-term coefficients. The network was provisionally configured with a single hidden layer, using magnetic interference magnitudes as training labels. Validation was conducted with labeled data to compensate for maneuver-induced magnetic interference. Figure 9b compares the magnetic compensation performance of 18-features versus the 34-features magnetic interference predictions.

As evidenced in Table 1, the introduction of dynamic-enhanced terms increased the improvement ratio by 87.49% and 65.15% for the linear regression and neural network methods, respectively. Furthermore, under identical conditions, the neural network approach outperformed linear regression in magnetic interference compensation efficacy. These findings verify the feasibility of the proposed dynamic-enhanced model.

Proceeding to neural network compensation model and algorithm design, given that incorporating dynamic-enhanced terms enhanced the IR, the 34-term coefficients from the modified T-L model were selected as neural network inputs, with the magnetic interference magnitude as the output metric. Crucially, the limited aeromagnetic dataset rendered deep neural networks unsuitable—their excessive complexity relative to data volume would induce severe overfitting and training non-convergence. We therefore employed a shallow single-hidden layer network. We compared training outcomes across neural networks with varying layer counts, as summarized in Table 2. Optimal network parameters were determined using three key metrics: IR, the MSE of test values, and the correlation coefficient R between the actual and predicted magnetic interference values. The MSE and the correlation coefficient R are given by the following formulas:

(21) $$MSE=\frac{1}{n}\sum_{i=1}^{n}{\left(y_{i}-\hat{y}\right)}^{2},$$

(22) $$R=\frac{\sum_{i=1}^{n}\left(y_{i}-\bar{y}\right)\left({\hat{y}}_{i}-\bar{\hat{y}}\right)}{\sqrt{\sum_{i=1}^{n}{\left(y_{i}-\bar{y}\right)}^{2}}\cdot \sqrt{\sum_{i=1}^{n}{\left({\hat{y}}_{i}-\bar{\hat{y}}\right)}^{2}}},$$

where $y_{i}$ denotes the actual value of the i-th sample; $\hat{y}$ represents the predicted value of the i-th sample; $\bar{y}$ is the mean of all actual values; and $\bar{\hat{y}}$ is the mean of all predicted values. A smaller MSE indicates closer proximity between predicted values and true values, signifying better predictive performance of the neural network. An R value closer to 1 suggests that the predicted values are more closely aligned with true values.

As evidenced in Table 2, single-hidden-layer networks demonstrated superior compensation performance and architectural efficacy compared to double/triple-layer configurations as the hidden layer depth increases. Consequently, the (34-12-1) single-layer topology outperformed alternative network parameterizations. Therefore, subsequent experiments adopted single-hidden-layer neural networks for training. Furthermore, variations in hidden node counts significantly impacted compensation efficacy. According to Equation (13), the node counts (7, 9, 11, 13, 15) were experimentally evaluated, with optimal parameters determined by maximizing the IR and correlation coefficient (R) while minimizing test mean squared error (MSE) between actual and predicted magnetic interference values.

As evidenced in Table 3, the configuration with nine hidden layer nodes achieved optimal performance: the IR peaked at 9.0657, the MSE was minimized at 0.0301, and the correlation coefficient R between the actual and predicted magnetic interference values reached 0.99115. This demonstrated exceptional prediction accuracy of magnetic interference, consequently indicating superior compensation efficacy. Therefore, the 9-node architecture was selected as the optimal architecture. Building upon this feasibility validation, a single-hidden-layer backpropagation neural network with nine neurons was ultimately adopted for subsequent magnetic interference compensation experiments.

**Figure 10:**
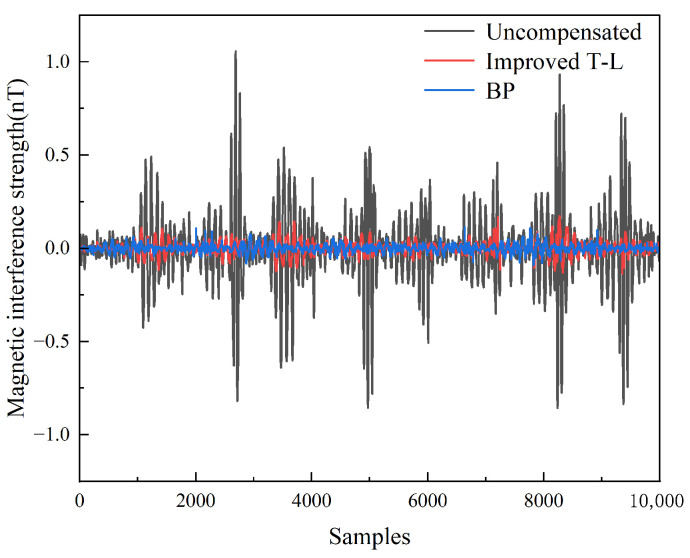
Compensation results for the improved T-L model and BP neural network.

Subsequently, leveraging the designed network architecture, experimental data were processed via the improved T-L model to generate 34-dimensional feature inputs (n × 34) for the BP neural network, with actual magnetic interference values (n × 1) serving as training labels. The network configuration employed a single hidden layer with nine neurons, as previously optimized. Compensation result based on this BP neural network is shown in Figure 10, while Table 4 quantitatively contrasts these outcomes against the least-squares method performance.

Through experiments, we found that both the BP neural network and the improved T-L model exhibited significant noise suppression effects, but the BP model demonstrated superior comprehensive performance. The uncompensated standard deviation (STD_n_) of the improved T-L model was 0.2013 nT, which decreased to 0.0368 nT after compensation, with an IR of 5.4739. The BP model showed strong fitting capability, further reducing the standard deviation to 0.0186 nT after compensation, achieving a high improvement rate of 11.7547. This indicated that the neural network method has obvious advantages in compensation accuracy.

**Figure 11:**
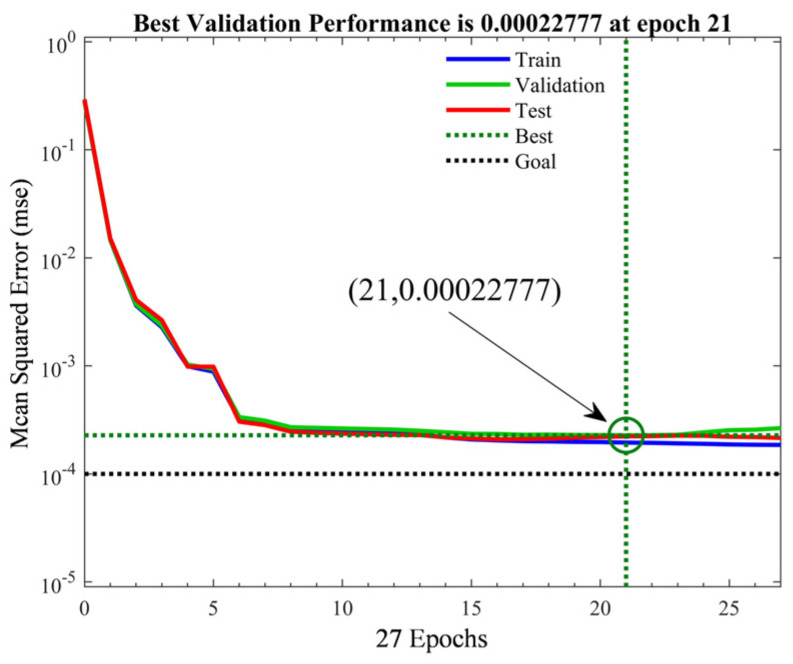
Performance of the BP neural network.

**Figure 12:**
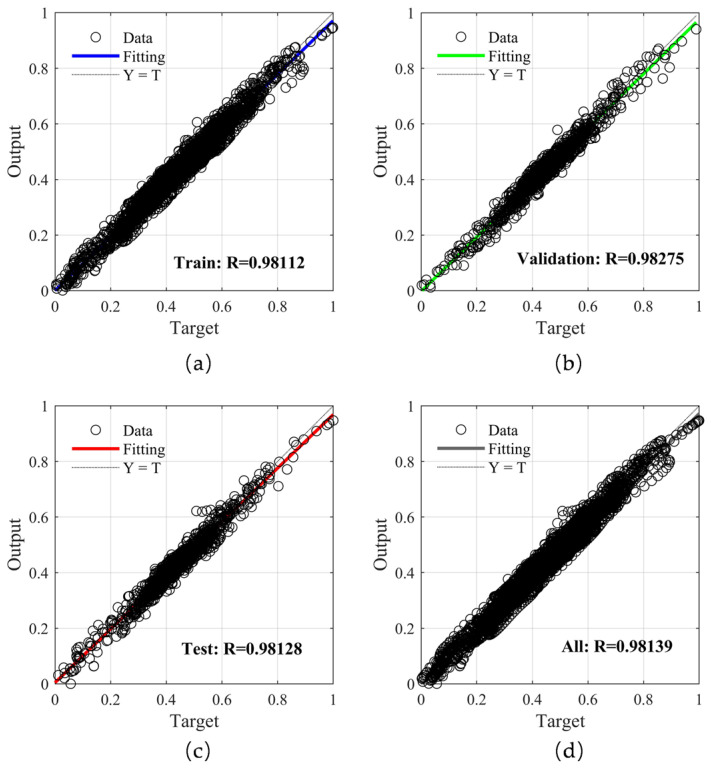
Linear regression results for the BP neural network data. (**a**) Train data; (**b**) Validation data; (**c**) Test data; (**d**) Overall data.

The training results for the BP neural network were evaluated as follows. As shown in Figure 11, the MSE showed a convergent trend with an increase in the number of iterations and reached the minimum at 21 iterations and 75 s when the BP neural network achieved optimal performance. The following figure presents the regression plots of the training set, validation set, test set, and all data relative to the network output in the BP neural network. As shown in Figure 12, the correlation coefficient R between the target values and the output values remained at approximately 0.98, indicating that the network can accurately learn the relationships between data and make accurate predictions. However, it was still evident that there is room for improvement in its generalization ability, which can be optimized.

**Figure 13:**
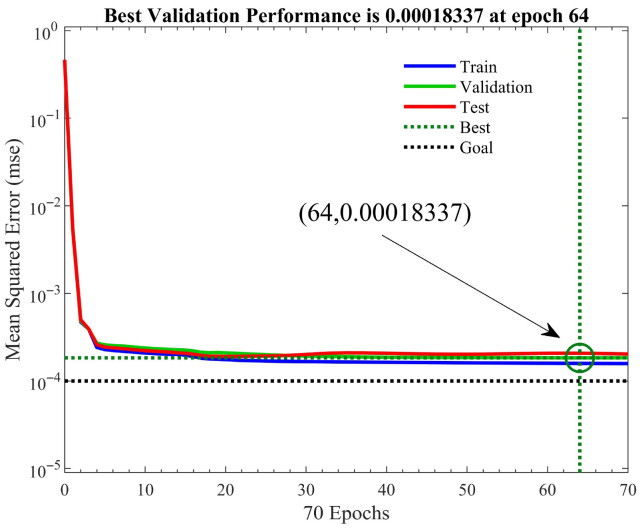
Performance of the GA-BP neural network.

**Figure 14:**
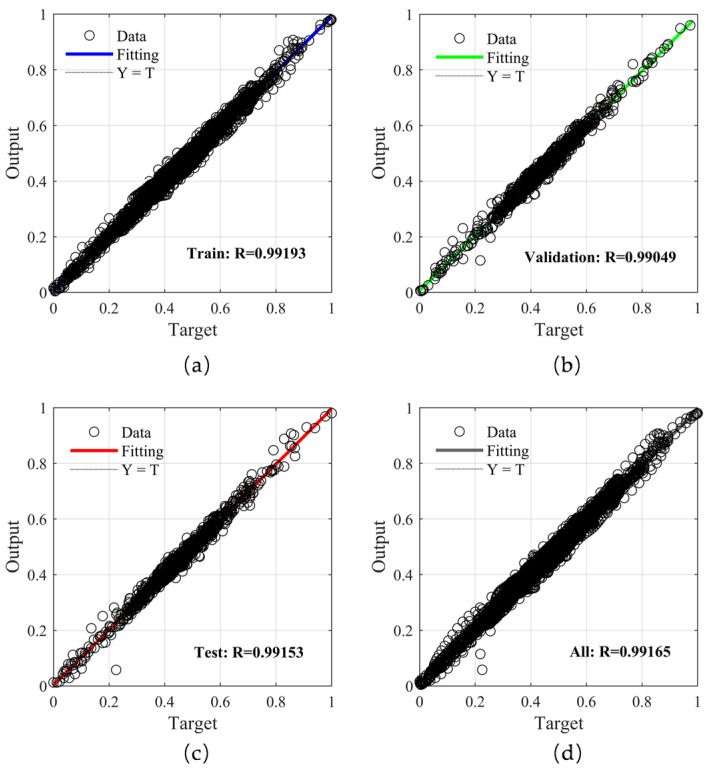
Linear regression results for the GA-BP neural network data. (**a**) Train data; (**b**) Validation data; (**c**) Test data; (**d**) Overall data.

Based on the above analysis of the BP neural network output results, the genetic algorithm was adopted to improve the BP neural network. As shown in Figure 13, we found that the optimized network reached the minimum MSE at 64 iterations and 142 s, while the unoptimized BP neural network reached the minimum MSE at 21 iterations and 75 s, and its MSE was higher than that of the optimized BP neural network. This indicated that without optimization, due to the random selection of weights and the use of gradient descent for updating, the network is prone to falling into a local optimum; this problem was effectively solved after optimization. As shown in Figure 14, it can be seen that after optimization with the genetic algorithm, the correlation coefficients between each group of data and the network output values were all above 0.99. Therefore, it can be concluded that the predicted performance of the network has been improved.

**Figure 15:**
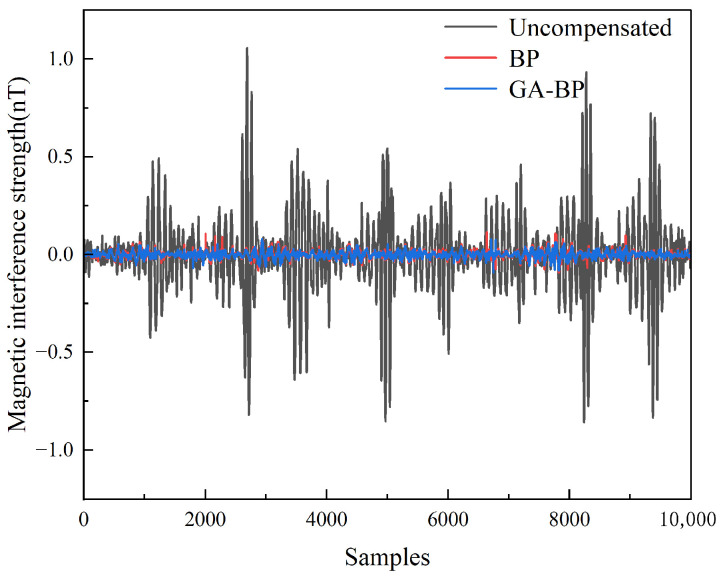
Compensation results for the BP neural network and GA-BP neural network.

**Figure 16:**
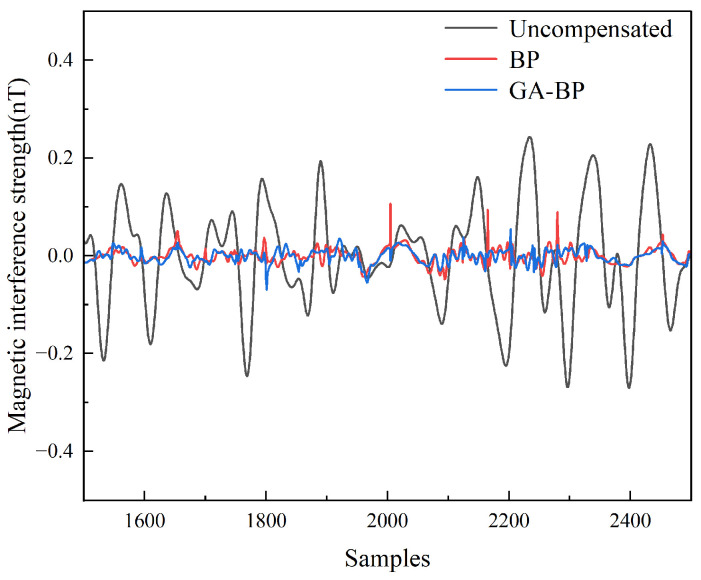
Local enlargement diagram of the compensation results for the BP neural network and GA-BP neural network.

Figure 15 shows a comparison between the compensation results for the optimized BP neural network with weight initialization via the genetic algorithm and those for the unoptimized BP neural network, with a partial enlarged view shown in Figure 16. The compensation results for the unoptimized BP neural network exhibited abnormal protrusions in certain areas, leading to deviations in the compensation outcomes. After optimization by the algorithm, it could be intuitively concluded that although GA-BP required longer training time than BP, GA-BP demonstrated improved compensation performance compared to the unoptimized BP neural network. As evidenced in Table 5, the 13.26% improvement in compensation results verifies the effectiveness of genetic algorithm optimization.

**Figure 17:**
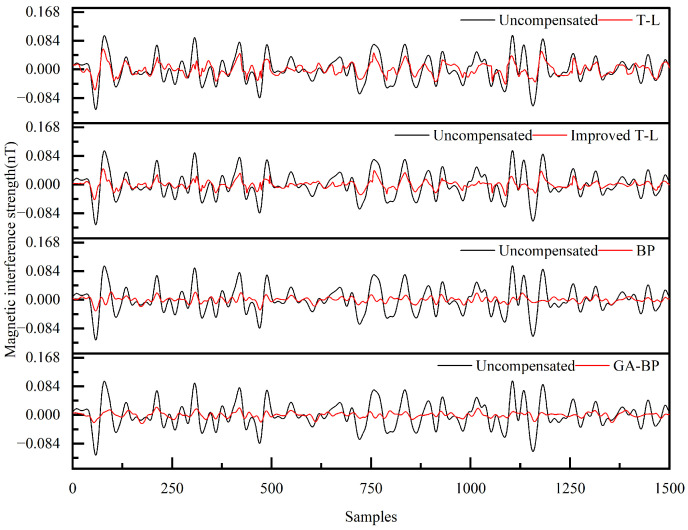
Compensation results for the four algorithms for Level Flight 1.

**Figure 18:**
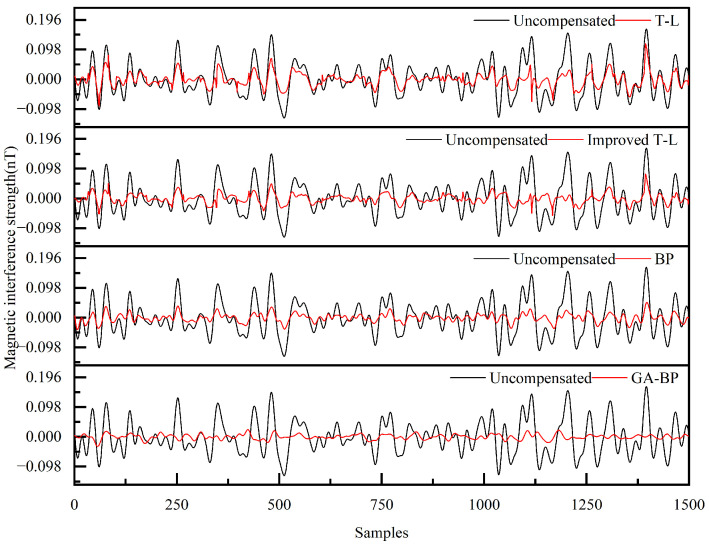
Compensation results for the four algorithms for Level Flight 2.

Although the magnetic interference compensation effect for the maneuvering actions of the UAV is relatively good, for magnetic detection tasks, the magnetic interference during the level flight phase needs to be optimized with priority. Since level flight is the core phase for data acquisition, there are differences between the steady-state interference in this phase and the transient interference excited by the maneuvering actions of the compensation loop. Therefore, it is necessary not only to compare the reliability of the magnetic interference compensation algorithms for the maneuvering actions but also, more importantly, to compare the reliability of these algorithms during the level flight phase. This directly determines the accuracy of the magnetic anomaly signals collected in magnetic detection tasks. Therefore, we conducted comparative experiments on the compensation of magnetic interference noise by different algorithms for the UAV under different flight conditions, aiming to eliminate the accidental deviations of single-group data and the risk of overfitting, and to ensure the reliability and universality of the results. The compensation results are shown in Figure 17 and Figure 18.

As evidenced in Table 6, comparative analysis of the level-flight magnetic compensation confirmed robust performance across all four algorithms, with each method effectively suppressing interference under varying operational conditions. The enhanced T-L model demonstrated superior compensation capabilities relative to its conventional counterpart, particularly during steady-state flight operations. Further improvement was achieved through the BP neural network, which outperformed both T-L variants in noise reduction efficacy. Most significantly, genetic algorithm optimization elevated the BP neural network performance substantially, yielding 32.21% and 28.70% accuracy gains in respective level-flight scenarios compared to non-optimized implementations. This hierarchical progression—from baseline T-L to the GA-BP neural network—established a clear performance gradient while validating the robustness of machine learning-enhanced compensation architectures.

## 4. Discussion

This study addresses limitations in existing airborne magnetic compensation models by proposing an intelligent machine learning-based approach. This method enhances the traditional T-L model through dynamic term expansion and neural network integration. The specifics are listed as follows:

Beginning with magnetic interference mechanisms, we extended the eddy-current component of the T-L model by incorporating Euler angles (roll, pitch, yaw) and their angular rates from the UAV’s inertial navigation system, along with relevant cross-terms. This dynamic-enhanced model demonstrated significantly improved compensation performance compared to the baseline T-L framework, as validated through comparative analysis.

The 34 expanded parameters were subsequently employed as input features for BP neural network training, with measured magnetic interference values serving as regression targets. This hybrid approach achieved superior compensation accuracy over conventional methods. To mitigate the BP neural network convergence instability caused by random weight initialization, we implemented GA optimization, which further enhanced the compensation precision.

While demonstrating clear advantages, this method presents opportunities for refinement:

- The dynamic enhancement currently focuses exclusively on eddy-current interference through kinematic parameters. Future work should incorporate detailed coupling analyses of additional interference sources.
- The GA’s computational demands necessitate feature-rich inputs for optimal network configuration. Future studies will explore automated architecture research given sufficient data availability.

While this study employed a shallow network, it is noteworthy that the exclusion of deep learning layers (DLL) was fundamentally driven by the problem’s moderate complexity and operational deployment requirements, not merely by dataset size. Future work may explore DLL architectures when addressing more sophisticated interference scenarios or multi-sensor fusion tasks.

## 5. Conclusions

The accuracy of UAV magnetic interference compensation critically determines the reliability of magnetic anomaly detection. Integrating established airborne compensation principles with magnetic interference mechanisms, this work introduced a dynamically enhanced T-L model. This extension incorporated Euler angles (roll, pitch, yaw) and the associated angular rates from the UAV’s inertial navigation system, along with their cross-terms, to expand the original coefficient space. Validation through comparative compensation analyses confirmed the efficacy of these dynamic enhancements. To address the inherent limitations of linear regression in noise fitting, we employed a BP neural network with the expanded 34-parameter T-L features as inputs and measured magnetic interference as training targets. This approach significantly improved compensation performance. Further mitigating BP neural network convergence instability caused by random weight initialization, GA optimization was implemented, with experimental results demonstrating its effectiveness. Comprehensive evaluation of the level-flight noise compensation across four algorithms revealed that neural network methods consistently outperform linear regression, with the GA-BP neural network achieving the highest compensation accuracy.

## Figures and Tables

**Table 1:** Comparison of feasibility validation results.

Verification Method	Model	STDu (nT)	STDc (nT)	IR
Linear regression	T-L model	0.2355	0.0806	2.9195
	Improved T-L model		0.0430	5.4739
Neural networks	18 input features	0.2355	0.0429	5.4893
	34 input features		0.0259	9.0657

**Table 2:** Training results with different numbers of hidden layers.

Hidden Layers	12	12-6	12-6-12
IR	7.8155	6.7678	7.6022
MSE	0.0345	0.0392	0.0348
R	0.98920	0.98609	0.98902

**Table 3:** Training results with different node counts.

Number of Nodes	7	9	11	13	15
IR	7.274	9.0657	8.0988	7.8082	7.6728
MSE	0.0376	0.0301	0.0332	0.0346	0.0353
R	0.98722	0.99115	0.98943	0.98915	0.98871

**Table 4:** Evaluation indicators for the compensation results for the improved T-L model and BP neural network.

Model	STD_u_ (nT)	STD_c_ (nT)	IR
Improved T-L model	0.2013	0.0368	5.4739
BP		0.0186	11.7547

**Table 5:** Evaluation indicators for the compensation results for the BP neural network and GA-BP neural network.

Model	STD_u_ (nT)	STD_c_ (nT)	IR
BP	0.2013	0.0186	11.7547
GA-BP		0.0164	13.3129

**Table 6:** Evaluation indicators for the compensation results for the four algorithms under different level flights.

Flight	Model	STD_u_ (nT)	STD_c_ (nT)	IR
Level Flight 1	T-L	0.0357	0.0122	2.9260
	Improved T-L		0.0109	3.2672
	BP		0.0079	4.4715
	GA-BP		0.0060	5.9122
Level Flight 2	T-L	0.0558	0.0235	2.3771
	Improved T-L		0.0160	3.4801
	BP		0.0133	4.2703
	GA-BP		0.0102	5.4967

## Data Availability

Due to the specificity of the data, please contact the corresponding author for access.
